# A Sensitive Homecage-Based Novel Object Recognition Task for Rodents

**DOI:** 10.3389/fnbeh.2021.680042

**Published:** 2021-06-11

**Authors:** Jessica I. Wooden, Michael J. Spinetta, Teresa Nguyen, Charles I. O’Leary, J. Leigh Leasure

**Affiliations:** ^1^Department of Psychology, University of Houston, Houston, TX, United States; ^2^Department of Psychology, Seattle University, Seattle, WA, United States; ^3^Department of Biology and Biochemistry, University of Houston, Houston, TX, United States

**Keywords:** novelty recognition, memory, exploration, odor, sex differences, ethological relevance

## Abstract

The recognition of novel objects is a common cognitive test for rodents, but current paradigms have limitations, such as low sensitivity, possible odor confounds and stress due to being performed outside of the homecage. We have developed a paradigm that takes place in the homecage and utilizes four stimuli per trial, to increase sensitivity. Odor confounds are eliminated because stimuli consist of inexpensive, machined wooden beads purchased in bulk, so each experimental animal has its own set of stimuli. This paradigm consists of three steps. In Step 1, the sampling phase, animals freely explore familiar objects (FO). Novel Objects (NO1 and NO2) are soiled with bedding from the homecage, to acquire odor cues identical to those of the FO. Steps 2 and 3 are test phases. Herein we report results of this paradigm from neurologically intact adult rats and mice of both sexes. Identical procedures were used for both species, except that the stimuli used for the mice were smaller. As expected in Step 2 (NO1 test phase), male and female rats and mice explored NO1 significantly more than FO. In Step 3 (NO2 test phase), rats of both sexes demonstrated a preference for NO2, while this was seen only in female mice. These results indicate robust novelty recognition during Steps 2 and 3 in rats. In mice, this was reliably seen only in Step 2, indicating that Step 3 was difficult for them under the given parameters. This paradigm provides flexibility in that length of the sampling phase, and the delay between test and sampling phases can be adjusted, to tailor task difficulty to the model being tested. In sum, this novel object recognition test is simple to perform, requires no expensive supplies or equipment, is conducted in the homecage (reducing stress), eliminates odor confounds, utilizes 4 stimuli to increase sensitivity, can be performed in both rats and mice, and is highly flexible, as sampling phase and the delay between steps can be adjusted to tailor task difficulty. Collectively, these results indicate that this paradigm can be used to quantify novel object recognition across sex and species.

## Introduction

It has long been noted that rats interact with a novel object more than they interact with an object they have previously been exposed to (Berlyne, 1950). This natural tendency led to the creation of the novel object recognition test (NOR) (Ennaceur and Delacour, 1988), a paradigm commonly used in rodents to assess both recognition memory for familiar objects and preference for novel objects. Compared to other tests of recognition memory, NOR is desirable because it requires no reinforcement or punishment to motivate behavior and does not require prolonged training before it can be performed. It also relies on a rodent’s natural inclination to explore its environment, and to approach and interact with objects that hold novelty value (Renner, 1990; Bevins and Besheer, 2006). A major advantage of the task is that it can be used in both rats and mice (Agarwal et al., 2020; Chang et al., 2020). Current iterations of the NOR paradigm are widely used to study memory (Cole et al., 2020), synaptic plasticity (Lee et al., 2020), impairment and/or recovery of function in brain disease (Grayson et al., 2015), TBI (Amoros-Aguilar et al., 2020), stress (Brivio et al., 2020; Glushchak et al., 2021), aging (Amirazodi et al., 2020), sleep (Shahveisi et al., 2020), autism (Batista et al., 2019; Gandhi and Lee, 2020), and epigenetics (Ellis et al., 2020; Sadat-Shirazi et al., 2020).

In a prototypical paradigm, the animal is first exposed to two of the soon-to-be familiar objects (“sampling phase,” A + A), and then returned to its home cage for a retention period. In the second phase, the animal is exposed to one familiar object and one novel object (“test phase,” A + B). This novel object may differ from the familiar object in shape, size, color, or any number of attributes. The amount of time the rodent spends exploring the novel object is then compared to the amount of time spent exploring the familiar object in the test phase [for examples of this procedure, see Ennaceur and Delacour (1988), Akkerman et al. (2012)]. When the animal spends more time with the novel object, at levels significantly above chance, we conclude that it has discriminated between the objects based on features that it is accustomed to in the familiar object, and features that it detects as unfamiliar in the novel object.

The many advantages of currently used NOR paradigms are offset by at least four significant limitations. First, they take place in an open field or specially-designed chamber (such as a Y-maze) (Hornoiu et al., 2020), to which the animal must become accustomed. This increases handling stress (during transfer from the home cage into the testing chamber) as well as novelty stress and the potential for distraction toward aspects of the chamber and away from the stimuli, thereby reducing interaction with stimuli (Ameen-Ali et al., 2015). While some have conducted NOR in the home cage (Kemppainen et al., 2015), this approach is not widely used. Second, current NOR paradigms generally utilize only two objects at a time to determine novelty recognition. Some versions of the task, such as the object-in-place task meant to assess spatial novelty, use more than two stimuli, but novel object tasks typically do not. This necessitates that an animal spends a lot longer with the novel stimulus to make it over 50% (chance level exploration) (Ennaceur, 2010). Third, detection of a novel object capitalizes on spontaneous rodent behavior, and therefore many of the current novelty preference paradigms utilize only one trial per day in order to maintain spontaneity. This low number of trials is a source of variability and decreases the amount of possible data, thereby increasing the necessary experimental *n* [for review see (Ameen-Ali et al., 2015)]. Fourth, there is no standardization of the objects used as stimuli. Functional properties of objects can have a major influence on a rodent’s baseline interest in an object and can be affected by whether the object is affixed or can be moved (Heyser and Chemero, 2012). Stimuli may also be overly large, or otherwise difficult for a rodent to climb on, pick up, manipulate, move around or chew, all of which are ways that they naturally show interest in an object (Dere et al., 2007). These differences can be difficult to predict or measure and could theoretically influence task difficulty [for images of various object used see (Gulinello et al., 2019)]. Moreover, stimuli are typically re-used between animals, necessitating sanitization between trials and between animals, in order to eliminate scent-marking and other odor cues. Ironically, use of a sanitizing agent itself introduces a powerful odor, one that experimental animals may find overwhelming, aversive, or both (Gulinello et al., 2019). These issues are of particular concern in high-throughput testing situations or in test areas with low ventilation.

Herein, we introduce a paradigm that addresses methodological issues concerning sensitivity, number of trials, and test environment. It also introduces the use of mass-produced, machined wooden stimuli, which are disposable, thus eliminating confounding odor cues. The paradigm includes an optional second test phase, in which animals are presented with a second novel object (NO2) in addition to the original novel object (NO1). We present data obtained from neurologically intact rats and mice of both sexes.

## Materials and Methods

### Animals

Long–Evans rats (females *n* = 18, males *n* = 15) weighing 200–400 g, and C57Bl/6J mice (females *n* = 16, males *n* = 16) were used. Rats were ordered from Envigo and were approximately 70 days old during testing, while mice were bred in-house and tested at approximately 80 days old. Animals were group-housed (rats three per cage, mice 5 per cage), maintained on a reversed light/dark cycle (9 A.M. off/9 P.M. on), and given access to food and water *ad libitum*. Our facility uses OptiMice and OptiRat housing from Animal Care Systems. The dimensions of each mouse cage are 13.5” (34.3 cm) L × 11.5” (29.2 cm) W (front) × 6.1” (15.5 cm) H, while the dimensions of each rat cage are 14” (35.6 cm) L × 19.1” (48.5 cm) W × 8.6” (21.8 cm) H. All animals remained group-housed throughout the experiment, and rats were housed with Alpha-Dri bedding while mice were housed with Alpha-Pad bedding. Rats and mice were free to create nests out of the bedding material and interact with their food and water dispensers, but no enrichment objects were introduced to the cage. Both rats and mice were fed Picolab Rodent Diet 20 5053 and all animals were gently handled to familiarize them with the researchers. Testing occurred during the animals’ dark cycle. All animal care and experimental procedures were approved by the University of Houston’s Institutional Animal Care and Use Committee.

### Preference Testing

In order to ensure that animals do not have an innate preference for any of the shapes we would be using, we first conducted a preference test with separate groups of naive rats and mice (age-matched to those used in the rest of the study) to assess natural exploration of the sphere, cube and beehive shapes. Female (*n* = 17) and male (*n* = 39) rats and female (*n* = 14) and male (*n* = 8) mice were given one 60-s trial during which they were presented with all three objects, and time spent exploring each one was recorded (using the methods described below).

### Novel Object Recognition Task

#### Stimuli

**FIGURE 1:**
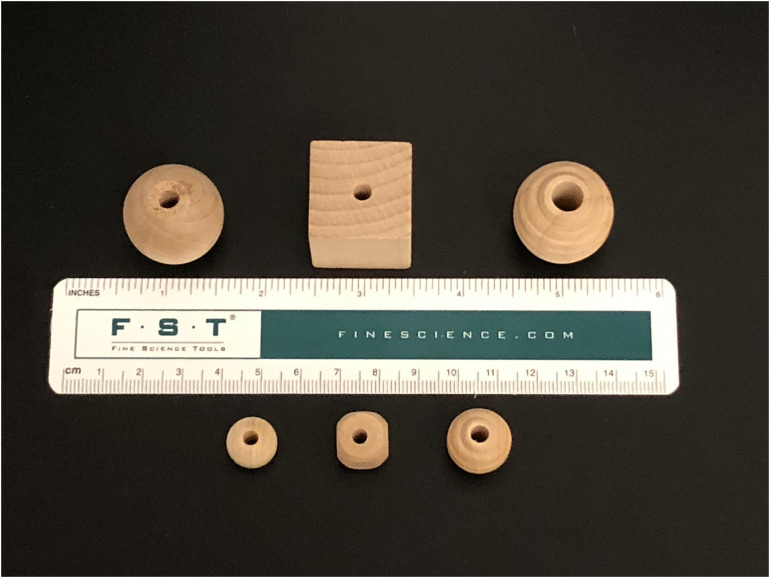
Machined wooden beads used as stimuli for rats **(top)** and mice **(bottom)**.

All stimuli were mass-produced, machined wooden shapes available from Woodworks Ltd.^1^. Stimuli were the same shape for rats and mice, but the mouse stimuli were smaller (see Figure 1). Spherical beads (rat: model #BE1090; mouse: model #BE1030), served as familiar objects (FO) for all trials. The novel objects were a cube shape (rat: model # BE3060; mouse: model #BE5050) and a beehive shape (rat: model # BE6090; mouse: model #BE6010). All objects had holes drilled through the center, and were light enough for the animals to pick up and manipulate. Each animal had its own set of stimuli, eliminating the need for sterilization procedures that could introduce unintended odor cues. Using mass-produced, machined, disposable wooden stimuli offers a number of advantages. First, being made of wood, the stimuli are light and can be easily manipulated by the animal, enabling them to engage in natural behaviors such as chewing or moving the objects around the cage. In particular, the rats that we tested appeared motivated to interact with the stimuli for a considerable amount of time, sometimes even picking them up in their teeth and moving them around the cage, a functional advantage of the objects as they allow for cross species comparison (Blaser and Heyser, 2015) and greater exploration by mice (Heyser and Chemero, 2012). Second, these stimuli are available in a wide variety of sizes, so larger ones can be used for testing rats and smaller ones can be used for testing mice. Third, because the stimuli are disposable, confounding odor cues are eliminated, as novel objects are never used more than once, eliminating the potential for scent marking and the need to sanitize objects between trials.

#### Step 1: Sample Familiar Objects

**FIGURE 2:**
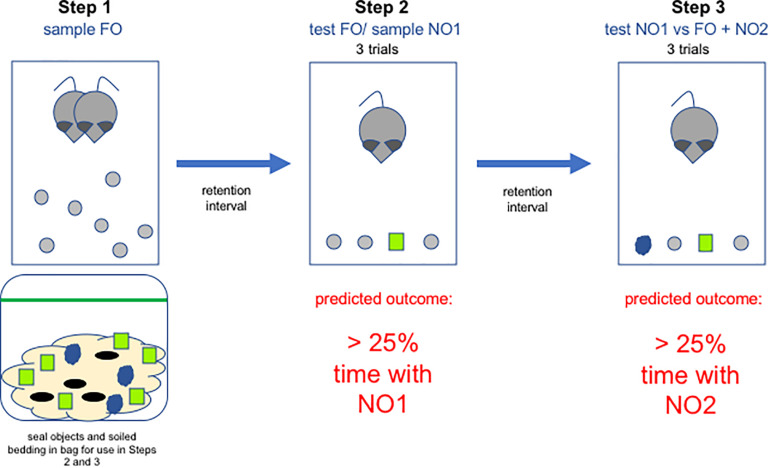
Diagram of the steps in the paradigm all of which take place in the home cage. In Step 1, rats become accustomed to the presence of wooden objects in the homecage. These will serve as the familiar objects (FO) in Steps 2 and 3. In addition, soiled bedding from the home cage is sealed in a bag along with novel objects for use in Steps 2 and 3. FO are removed and then, after a retention interval, reintroduced along with a novel object (NO1) in Step 2. Objects are again removed and following a second retention interval, Step 3 is performed, in which 2 FO are placed in the cage along with NO1 and a second novel object (NO2).

In the present set of experiments, cages were cleaned approximately 2 days before the start of the experiment, and animals were allowed to get used to the FO overnight. Three spherical beads per animal were introduced into each home cage overnight before the NOR test began, so that they could acquire the odor of the animals, and also so that the animals would become familiar with their size and shape. One or two extra beads were placed in each cage in case the animals were to gnaw a bead, making it distinctive and thus unusable. Which of the remaining shapes (cube or beehive) served as the first (NO1) or second (NO2) novel object was counterbalanced. For each animal, 6 NO1 and 3 NO2 were placed into a sealed plastic bag along with a handful of soiled bedding from the cage. This was done so that the stimuli would acquire the scent of the home cage, and could not be identified as novel based on scent. Each bag was carefully labeled to ensure that each individual subject was only exposed to their individual set of stimuli and that those stimuli were not exposed to any other animal’s bedding. For a graphical representation of the task, please see Figure 2. For a short video, please see Supplementary Material.

#### Step 2: Test FO and Sample NO1

The day after FO were placed in the homecage, they were removed for 1 h and three were placed in each animal’s sealed plastic bag along with the novel objects. Animals were transported to a darkened testing room and acclimated for 15 min before testing began under red light illumination. Testing occurred in the home cage, so just before the trials began, cage mates were removed and placed into a separate clean cage. Next, three spherical beads were removed from the subject’s sealed plastic bag and placed in the cage along with one of the NO1 objects (either cube or beehive, counterbalanced). Stimuli were placed in a row, parallel to the front end of the home cage, with the holes facing up. Animals were exposed to these four objects for three 1-min trials with 1-min inter-trial intervals during which time the objects were removed from the cage. Each animal stays in their home cage for the entire testing session (all three trials and the two 1 min inter-trial-intervals, approximately 5 min). The focus was to assess exploration of NO1 in the presence of three familiar objects. To eliminate scent-marking as a confound, NO1 was discarded after each trial and replaced by a “fresh” NO1 taken from the sealed plastic bag. The spatial location of the stimuli was randomized between trials to ensure that any inherent place preference did not artificially increase time spent with a particular stimulus. The latency to approach any of the four stimuli was recorded using a stopwatch, and this first approach initiated the 1-min trial. Exploration time for each of the four objects was recorded using ODLog (Macropod Software) and an external keypad that had four coded keys (one for each stimulus). This allowed a single experimenter to monitor and record animals responses to each of the four stimuli in real-time. An animal was determined to be exploring an object if it’s snout, vibrissae or front paws were in contact with the object. After the third trial, the three spherical FO were placed back into the home cage overnight. This was done in order to re-familiarize the animals with the objects, so that the focus of Step 3 would be the distinction between NO1 and the second novel object (NO2). The experimenter donned a fresh pair gloves before proceeding to test the next cage, to avoid cross-contaminating objects with the smell of another cage.

#### Step 3: Test NO1 Versus FO + NO2

Step 3 was performed 24 h after Step 2 on a randomly selected subset of the animals. For this phase of the task, animals were presented with two FO, one NO1 and one unfamiliar novel object (NO2), all of which had been sealed in a bag with the animal’s home cage bedding. The focus was to assess exploration of NO2 in the presence of NO1 and two familiar objects. Three 1-min trials were administered to each animal and time spent exploring each of the four stimuli recorded, as described above.

### Data Analysis

**FIGURE 3:**
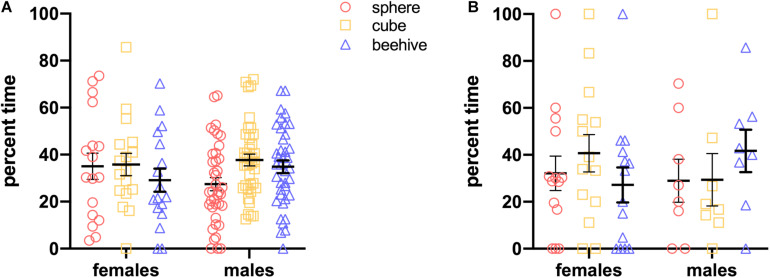
In an initial test, there was no grouped preference for any of the three objects in naïve rats **(A)** or mice **(B)**. Particularly among mice, however, individuals ignored one or more objects.

Data were analyzed using IBM SPSS Statistics software v26. Raw time spent exploring each object was translated into a percentage time by dividing mean time for each object by total mean exploration time for all objects. Percentage time is calculated for both Steps 2 and 3. The formula for the calculation for Step 2 is: NO1/(FO1 + FO2 + FO3 + NO1). For Step 3, using NO1 as an example, the formula for this calculation is NO1/(FO1 + FO2 + NO1 + NO2). This formula is a slight modification of the commonly used Recognition Index (RI) formula (Mumby et al., 2002; Antunes and Biala, 2012; Schoberleitner et al., 2019) with the addition of more stimuli. Since they have the opportunity to explore four objects in a given trial, an animal performing at chance would explore each object for 25% of their total time. An individual animal’s trial was excluded from analysis if they failed to explore NO1 on Step 2 or either NO1 or NO2 on Step 3. In order to assess preference for a novel object, we reasoned that an animal must contact that novel object. The initial preference testing we conducted in naïve rats and mice showed that it was possible that an animal would fail to contact one of the presented stimuli on a given trial (see Figure 3). Ignoring one or more objects was particularly prevalent in mice, some of which spent the entire trial exploring only one of the three stimuli presented. We therefore decided that our test phases would consist of three trials and that analyses would be performed on each animal’s data from trials in which they contact NO1 and a FO in Step 2. Similarly in Step 3 for trials in which animals contact NO1 and NO2. Thus, in the event that an animal ignored one or more novel stimuli on a given trial, that trial would not be included in the analysis. All trials that reached these simple performance criteria were included, thus in the current series of tests, only one male rat was excluded from the analysis of Step 3 data. For all statistical analyses on Steps 2 and 3, one-way ANOVA’s were conducted to determine if there were differences in the percentage time exploring objects, with an alpha level of 0.05 set to determine significance. Homogeneity of variances tests were conducted to determine what *post hoc* corrections were appropriate. Habituation to NO1 across trials in Step 2 was assessed using an independent samples *t*-test. Linear regression analyses were performed to determine whether exploration of NO1 on Trial 3 of Step 2 was predictive of exploration of NO1 on Trial 1 of Step 3. *T*-tests were also performed to explore sex and species differences in overall time spent exploring objects as well as average latency to approach objects.

## Results

### Preference Testing

We exposed naive rats and mice of both sexes to the stimuli. As shown in Figure 3, on a population level, rats and mice did not display a preference for any of the objects, as percent time spent with each was similar. For rats, two-way ANOVA showed no main effect of Sex [*F*(1,162) = 0.00710, *p* = 0.998] or Object [*F*(2,162) = 1.294, *p* = 0.187] and no significant interaction [*F*(2,162) = 1.297, *p* = 0.277]. Similarly, for mice, two-way ANOVA showed no main effect of Sex [*F*(1,60) < 0.000, *p* > 0.999] or Object [*F*(2,60) = 0.151, *p* = 0.860] and no significant interaction [*F*(2,60) = 1.117, *p* = 0.334]. Nonetheless, particularly with the mice, there were some animals that spent no time at all with one or more of the objects. This is noteworthy because it indicates that there is a distinct possibility that at least one experimental animal will ignore a novel object on one or more trials. As noted above, this is the rationale for performing three trials in each of the test phases.

### Novel Object 1: Rats

**FIGURE 4:**
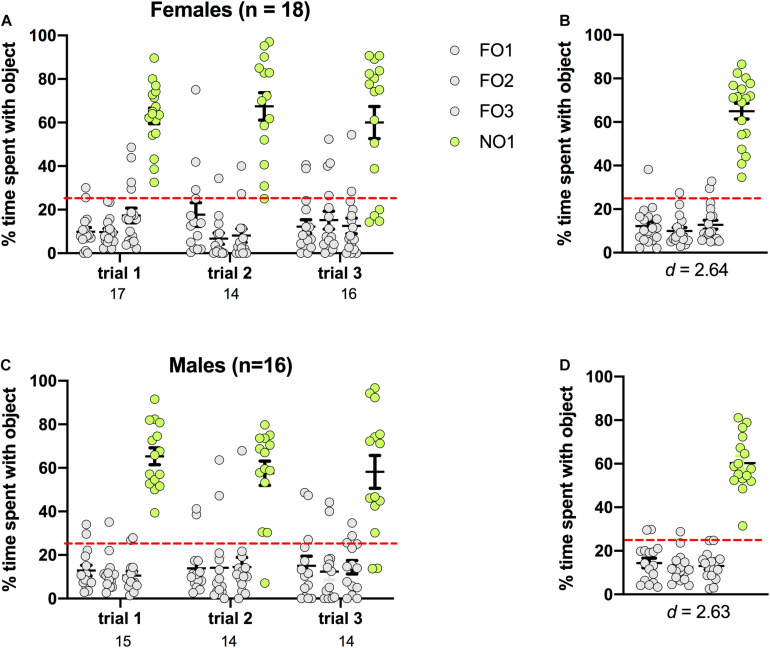
Rats of both sexes showed a significant preference for NO1 over the three familiar objects. Both females **(A,B)** and males **(C,D)** spent a greater percentage time than chance (dotted red line) exploring NO1. Panels **(B,D)** show the data averaged across all three trials. Numbers below the X-axis in panels **(A,C)** indicate the number of animals that contacted NO1 during each trial. Effect sizes (Cohen’s *d*) are indicated below the X-axis in panels **(B,D)**, and were calculated as a marker of practical significance for exploration differences between NO1 and FO1.

Preference for NO1 was indicated by significantly more percentage time spent exploring NO1 than familiar objects across three trials of Step 2. As shown in Figure 4, rats of both sexes displayed a distinct preference for NO1 versus the three familiar objects. In females, there was a significant main effect of Object [*F*(3,184) = 116.366, *p* < 0.001, η^2^ = 0.65], and *post hoc* Games-Howell corrected comparisons revealed that they spent significantly more time exploring NO1 than all three familiar objects (*p* < 0.01 for all comparisons). Female rats also spent a greater percentage time than chance (25%) exploring NO1 than each familiar object. There were no differences in the exploration time between familiar objects. Results were similar in males, with a significant main effect of Object [*F*(3,168) = 98.808, *p* < 0.001, η^2^ = 0.64]. *Post hoc* Games-Howell corrected comparisons revealed male rats spent significantly more time exploring NO1 than all three familiar objects (*p* < 0.01 for all comparisons). Male rats also spent a greater percentage time than chance (25%) exploring NO1 than each familiar object. There was no difference in the exploration time between familiar objects. As there were no differences in the exploration time between familiar objects, Cohen’s *d* was calculated as a measure of practical significance for the difference in percent time spent exploring NO1 and FO1, revealing large effect sizes in both females *d* = 2.64, and males *d* = 2.63 (see Figure 4). Interestingly, female rats spent less overall time exploring objects than males *t*(32) = -2.281, *p* = 0.029 (see Table 1 for the difference in mean time spent exploring all objects in Step 2). Independent samples *t*-tests revealed no change in exploration of NO1 between trials 1 and 3 in females *t*(19.804) = 0.349, *p* = 0.731, or males *t*(17.138) = 1.261, *p* = 0.219. Finally, as part of the NOR paradigm, we started a timer to record latency to first contact of any object. We assessed whether there were sex differences in latency and discovered that there were no differences in the average latency to contact objects between female and male rats in Step 2 *t*(32) = 1.242, *p* = 0.223 (see Table 1 for the differences in average latency to approach objects in Step 2).

**TABLE 1:** Step 2 object exploration data.

**Sex/species**	***n***	**Mean time FO1**	**Mean time FO2**	**Mean time FO3**	**Mean time NO1**	**Total time all objects**	**Average latency**
Female rats	18	1.0 + 0.3	0.8 + 0.2	1.3 + 0.4	8.8 + 2.3	30.9 + 4.6*	20.2 + 5.9
Male rats	15	1.9 + 0.4	2.0 + 0.7	2.1 + 0.6	13.4 + 3.2	52.3 + 8.4	11.6 + 3.0
Female mice	16	0.7 + 0.2	0.6 + 0.1	0.7 + 0.1	5.9 + 1.5	16.6 + 3.4*	18.4 + 4.0
Male mice	16	0.8 + 0.1	0.6 + 0.1	1.2 + 0.4	9.4 + 2.8	27.5 + 6.3	12.1 + 2.0

*p < 0.05 significantly different from males of same species.

### Novel Object 1: Mice

**FIGURE 5:**
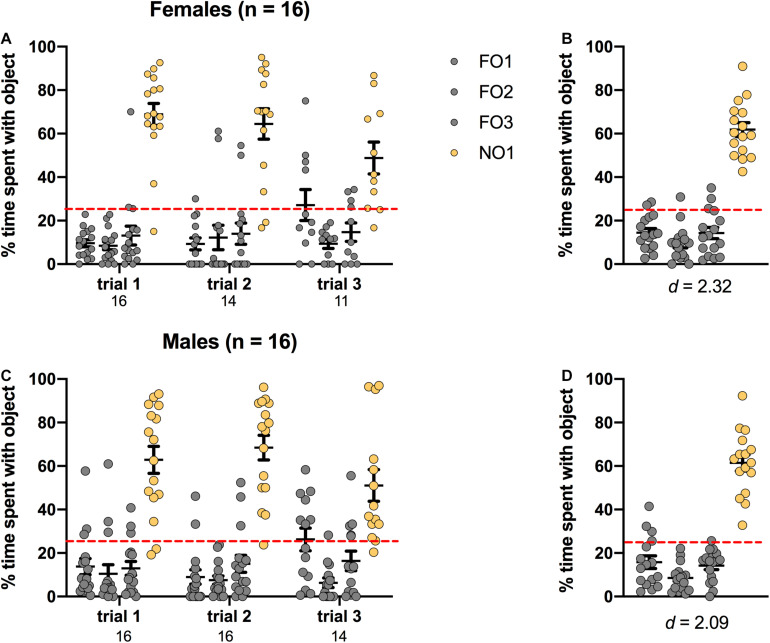
Mice of both sexes showed a significant preference for NO1 over the three familiar objects. Both females **(A,B)** and males **(C,D)** spent a greater percentage time than chance (dotted red line) exploring NO1. Panels **(B,D)** show the data averaged across all three trials. Numbers below the X-axis in panels **(A,C)** indicate the number of animals that contacted NO1 during each trial. Effect sizes (Cohen’s *d*) are indicated below the X-axis in panels **(B,D)**, and were calculated as a marker of practical significance for exploration differences between NO1 and FO1.

Preference for NO1 was indicated by significantly more percentage time spent exploring NO1 than familiar objects across three trials of Step 2. As shown in Figure 5, mice performed similarly to rats, in that both sexes displayed a distinct preference for NO1 versus the three familiar objects. In females, there was a significant main effect of Object [*F*(3,160) = 77.330, *p* < 0.001, η^2^ = 0.59], and *post hoc* Games-Howell corrected comparisons revealed that they spent significantly more time exploring NO1 than all three familiar objects (*p* < 0.01 for all comparisons). Female mice also spent a greater percentage time than chance (25%) exploring NO1 than each familiar object. There were no differences in the exploration time between familiar objects. Male mice performed similarly, with a significant main effect of Object [*F*(3,180) = 84.600, *p* < 0.001, η^2^ = 0.59]. *Post hoc* Games-Howell corrected comparisons revealed male rats spent significantly more time exploring NO1 than all three familiar objects (*p* < 0.01 for all comparisons). Male mice also spent a greater percentage time than chance (25%) exploring NO1 than each familiar object. There was no difference in the exploration time between familiar objects. As there were no differences in the exploration time between familiar objects, Cohen’s *d* was calculated as a measure of practical significance for the difference in percent time spent exploring NO1 and FO1, revealing large effect sizes in both females *d* = 2.32, and males *d* = 2.09 (see Figure 5) Unlike the sex differences observed in rats, there were no differences in overall time spent exploring objects observed between female and male mice in Step 2 *t*(22.603) = -1.492, *p* = 0.149 (see Table 1 for the difference in mean time spent exploring all objects in Step 2). Independent samples *t*-tests revealed that while a change in exploration of NO1 between trials 1 and 3 was observed in females *t*(20) = 2.607, *p* = 0.017 this was not observed in males *t*(26) = 1.528, *p* = 0.139. An analysis of the average latency to contact objects revealed that there were no differences between female and male mice in Step 2 *t*(30) = 1.382, *p* = 0.177 (see Table 1 for the differences in average latency to approach objects in Step 2).

### Novel Object 2: Rats

**FIGURE 6:**
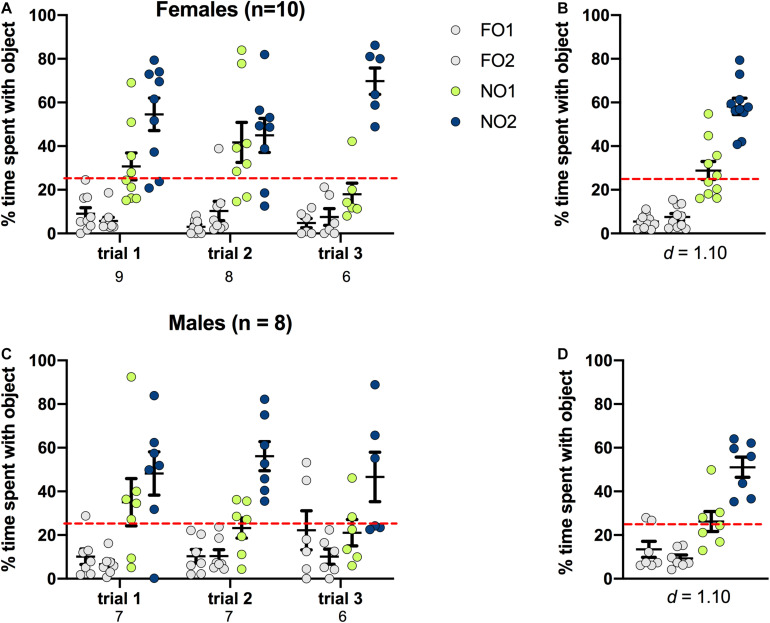
Rats of both sexes showed a significant preference for NO2 over NO1 and the 2 familiar objects. Both females **(A,B)** and males **(C,D)** spent a greater percentage time than chance (dotted red line) exploring NO2. Panels **(B,D)** show the data averaged across all three trials. Numbers below the X-axis in panels **(A,C)** indicate the number of animals that contacted NO1 during each trial. Effect sizes (Cohen’s *d*) are indicated below the X-axis in panels **(B,D)**, and were calculated as a marker of practical significance for exploration differences between NO1 and NO2.

As shown in Figure 6, rats of both sexes displayed a distinct preference for NO2 compared to either NO1 or the 2 FO. In females, there was a significant main effect of Object [*F*(3,88) = 46.316, *p* < 0.001, η^2^ = 0.61], and this was also the case for males [Object *F*(3,76) = 23.110, *p* = 0.001, η^2^ = 0.48]. *Post hoc* Games-Howell corrected comparisons revealed that rats of both sexes spent significantly more time exploring NO2 than NO1 or the FO (*p* < 0.01 for all comparisons). Cohen’s *d* was also calculated as a measure of practical significance for the difference in percent time spent exploring NO1 and NO2, revealing large effect sizes in both females *d* = 1.10, and males *d* = 1.10 (see Figure 6). Linear Regression Analyses were performed to determine whether exploration of NO1 on Trial 3 of Step 2 was predictive of exploration of NO1 on Trial 1 of Step 3. Results indicate that exploration of NO1 on Trial 3 of Step 2 was not predictive of subsequent exploration of NO1 on Trial 1 of Step 3 in females ß = 0.396, *t*(7) = 1.141, *p* = 0.292, or males ß = 0.349, *t*(4) = 0.746, *p* = 0.497. Moreover, rats of both sexes spent a greater percentage time than chance (25%) exploring NO2 than NO1 and familiar objects. There were no differences in the exploration time between familiar objects. As in Step 2, female rats spent less overall time exploring objects in Step 3 than males *t*(15) = -2.428, *p* = 0.028 (see Table 2 for the difference in mean time spent exploring all objects in Step 3). Similarly in Step 2, an analysis of average latency to contact objects revealed that there were no differences between female and male rats in Step 3 *t*(9.9) = 0.889, *p* = 0.39 (see Table 2 for the differences in average latency in Step 3).

**TABLE 2:** Step 3 object exploration data.

**Sex/species**	***n***	**Mean time FO1**	**Mean time FO2**	**Mean time NO1**	**Mean time NO2**	**Total time all objects**	**Average latency**
Female rats	10	0.7 + 0.2	0.9 + 0.3	4.0 + 1.0	9.5 + 3.2	36.9 + 5.6*	18.2 + 6.5
Male rats	8	2.0 + 0.5	1.7 + 0.5	6.6 + 2.9	10.2 + 2.6	60.2 + 8.3	12.3 + 1.5
Female mice	8	0.6 + 0.1	0.8 + 0.2	4.2 + 1.2	11.3 + 3.1	34.8 + 5.1	28.5 + 7.3
Male mice	8	0.4 + 0.1	0.5 + 0.1	4.0 + 1.2	6.6 + 2.0	30.4 + 6.5	19.7 + 7.3

*p < 0.05 significantly different from males of same species.

### Novel Object 2: Mice

**FIGURE 7:**
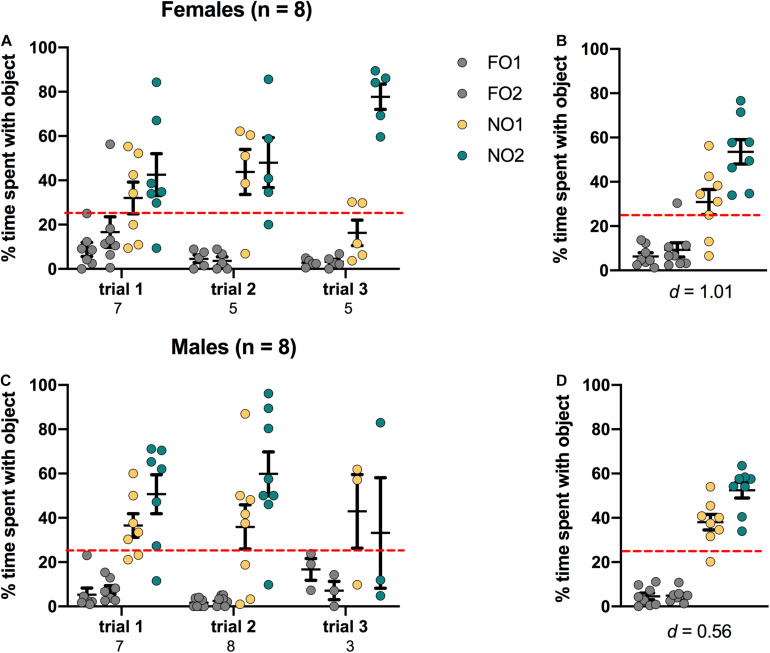
Mice of both sexes showed a significant preference for NO1 over the two familiar objects. Both females **(A,B)** and males **(C,D)** spent a greater percentage time than chance (dotted red line) exploring NO2. Panels **(B,D)** show the data averaged across all three trials. Numbers below the X-axis in panels **(A,C)** indicate the number of animals that contacted NO1 during each trial. Effect sizes (Cohen’s *d*) are indicated below the X-axis in panels **(B,D)**, and were calculated as a marker of practical significance for exploration differences between NO1 and NO2.

As shown in Figure 7, female mice displayed a distinct preference for NO2 compared to either NO1 or the 2 FO, with a significant main effect of Object [*F*(3,64) = 26.411, *p* < 0.001, η^2^ = 0.55]. *Post hoc* Games-Howell corrected comparisons revealed that female mice spent significantly more time exploring NO2 than NO1 or the FO (*p* < 0.05 for all comparisons). In males, there was a significant main effect of Object [*F*(3,68) = 27.873, *p* < 0.001, η^2^ = 0.55]. However, *Post hoc* Games-Howell corrected comparisons revealed that while male mice spent significantly more time exploring both NO2 and NO1 than the FO (*p* < 0.01 for both comparisons), they spent approximately an equal percentage of time with both novel objects. Cohen’s *d* was also calculated as a measure of practical significance for the difference in percent time spent exploring NO1 and NO2, revealing a large effect size in females *d* = 1.01, and a medium effect size (though not a statistically significant difference) in males *d* = 0.56 (see Figure 7). Linear Regression Analyses were performed to determine whether exploration of NO1 on Trial 3 of Step 2 was predictive of exploration of NO1 on Trial 1 of Step 3. Results indicate that exploration of NO1 on Trial 3 of Step 2 was not predictive of subsequent exploration of NO1 on Trial 1 of Step 3 in females ß = -0.897, *t*(2) = -2.868, *p* = 0.103, or males ß = -0.427, *t*(4) = -0.945, *p* = 0.398. As in Step 2, there were no differences in overall time spent exploring objects observed between female and male mice in Step 3 *t*(14) = 0.536, *p* = 0.60 (see Table 2 for the difference in mean time spent exploring all objects in Step 3). Finally, as seen in Step 2, an analysis of the average latency to contact objects revealed that there were no differences between female and male mice in Step 3 *t*(14) = 0.856, *p* = 0.41 (see Table 2 for the differences in average latency in Step 3).

## Discussion

This paper has two goals. The first is to introduce a paradigm of simple measures to reduce stress and improve sensitivity of the classic novel object recognition task. The entire paradigm takes place in the animal’s home cage, which provides a stable environment, with no changing contextual cues, thus ensuring that the only novelty is the test stimuli, not the chamber itself. Furthermore, testing in the home cage helps reduce environmental stress as the animal is accustomed to this environment. Importantly, testing in the home cage also eliminates the need to clean a testing chamber after trials and/or between animals, thus ensuring that the odor of disinfectant does not distract or distress the animals. Sensitivity is enhanced by utilizing four objects, establishing exploration above 25% as chance versus 50% as seen in classic NOR paradigms. The second goal of the manuscript is to present sample results from one possible iteration of our paradigm. Instead of manipulating various parameters, we chose to establish that our paradigm is broadly applicable to both rats and mice of both sexes, with relevant sex and species differences described below. Ultimately, we present this as a highly flexible paradigm that can be tailored for use in a wide variety of studies involving drug exposures, lesions, optogenetic manipulations and more. We present sample results from an iteration of this paradigm in which we used long (overnight) sampling phases with the FO, and short retention intervals (1 h). Ultimately, however, the paradigm is flexible, and the length of sampling and test phases easily altered to suit experimental needs. For example, task difficulty could be increased by shortening the (re)sampling phases with the FO and lengthening the retention intervals. In contrast, the task could be made easier with longer sampling phases and shorter retention intervals. Moreover, Step 3 is optional, and can be used if a challenging test of memory for NO1 is desired. Step 3 ultimately serves as two tests, one for memory of NO1, and a second, distinct novelty preference test for NO2. In the present iteration of the paradigm, animals were exposed to NO1 for a maximum of 180 s, yet rats of both sexes and female mice still spent significantly more time with NO2, indicating recognition of NO1. The preference for NO2 over NO1 is remarkable, given that the animals’ sum total sampling experience with NO1 occurred during the 3 trials of Step 2. Rats were easily able to detect the novelty of NO2, but it is possible that a longer delay between Steps 2 and 3 would make this more of a challenge for them. As a side note, we have pilot tested a Step 4, where we introduce NO3, but do not include it here as most rats showed task fatigue and declined to participate.

Although sampling phases, test phases, and inter-trial intervals can be adjusted to tailor task difficulty to experimental needs, two aspects of the paradigm are best held constant. The first is waiting to begin a trial until an animal contacts an object, because this ensures that each subject has the full minute in which to explore the stimuli. This results in lengthy exploration times, which is advantageous (see Tables 1, 2 for total exploration times with objects). The second aspect is the 1-min length of the trials. *T*-tests comparing percent exploration time between trials 1 and 3 were not significant in the majority of animals, indicating that 1-min trials do not produce habituation (female mice are the exception). We also performed linear regression analyses and determined that exploration of NO1 on Trial 3 of Step 2 was not predictive of exploration of NO1 on Trial 1 of Step 3. We therefore conclude that each trial can be viewed as a unique, 1-min opportunity to explore the novel objects. Indeed habituation is more likely to occur if the trials are lengthened as is the case in many NOR paradigms [see (Antunes and Biala, 2012) for review].

Our results generally support the use of three trials per step, and of analyzing percent time exploring each object. Although at the group level there was very little difference in outcome across trials, with animals spending significantly more than 25% time exploring the most recent novel object, there is inter-individual variability, and conducting three trials allows for the possibility that an animal does not explore one or more novel objects on a given trial. As we found in our preference testing (see Figure 3), this is particularly important for mice, as they can be prone to ignore an object. We eliminated from analysis any trial in which the animal did not contact NO1 and a FO on Step 2, or separately NO1 and NO2 on Step 3. As is common practice in NOR paradigms (Cole et al., 2019), this establishes a single performance criterion and minimizes bias because we cannot assume that an animal is exploring NO1 or NO2 preferentially in an individual trial if they do not contact both objects in Step 3. In Step 2, this would refer to NO1 and a familiar object. The number below the X axis in Figures 4–7 shows the number of animals (of the total n) that contacted the most recent novel object in each trial. A further advantage to using percent time is that it allows for individual differences in exploration without impacting interpretation of the results. As we have previously shown, some animals are considered to be “high explorers” and will spend a larger proportion of the test time interacting with novel stimuli (Spinetta et al., 2008). In contrast, others are “low explorers”, and will spend less time with novel stimuli, and may perhaps even fail to contact one of them. By calculating percent time spent with each stimulus, these natural differences can be controlled for, with the mean percentage time representing a universally reliable measure of exploration not biased by these differences in exploration. The calculation for percentage time presented here is based on the classic Recognition Index formula with the addition of two more objects NO1/(FO1 + FO2 + NO1 + NO2).

To ensure that our paradigm is broadly applicable to rodents, we tested neurologically intact rats and mice of both sexes. A sex difference was observed in time spent with the stimuli, with females of both species spending less time overall with the stimuli than males in Step 2. This difference, however, did not influence the primary outcome measure as rats and mice of both sexes detected the novel object in Step 2. In Step 3, there was again a sex difference in rats in time spent with the stimuli, again not influencing novelty detection in Step 3. For mice, however, only females preferred NO2 over NO1 in Step 3, though both males and females spent equal time with the stimuli. Overall this demonstrates that the observed sex differences in total exploration time did not influence novelty detection. In addition to a sex difference, we observed a species difference in Step 3. While rats of both sexes easily detected NO2 versus NO1, in mice, only females easily detected NO2 versus NO1. In contrast to rats and female mice, male mice on average preferred both novel objects equally, although notably, this effect was driven largely by trial 3, in which only 3 of 8 mice contacted NO2. It may be that six trials (across Steps 2 and 3) exhausts spontaneous novelty seeking behavior in male mice, and that Step 3 should be omitted for them, or perhaps that only one trial be utilized for that step.

Here we introduce and present sample results from a homecage-based paradigm that refines the classic novel object recognition task by improving sensitivity, standardizing objects used as stimuli and eliminating confounds (such as novelty of testing chamber and disinfection of stimuli), while at the same time maintaining cost-effectiveness and ease of administration. Because one the goals of this project was to ensure broad applicability across species and sexes, we did not test this paradigm against classic NOR paradigms. Nonetheless, our results indicate that this paradigm increases sensitivity over the classic NOR, because they show that rats and mice of both species explore the most recent novel object for approximately 60% of their time, demonstrating the high sensitivity of the task, as they have four objects to choose from (rather than 2). In other words, they demonstrate much higher than chance exploration (25%) compared to 2-stimuli paradigms (in which chance exploration is 50%). Our results also show that animals engaged in lengthy exploration times with the objects, suggesting that they were comfortable in the homecage environment and willing to engage with the stimuli, thus indicating that the goal of reducing stress (transfer and testing chamber novelty stress) was achieved.

Collectively, these results illustrate a homecage-based paradigm that can be used to quantify novel object recognition across sex and species. It maintains the strengths of the classic NOR tests while reducing stress, improving sensitivity and eliminating odor confounds. We believe that it will prove useful to a wide variety of researchers investigating brain health and function in rodent models, from basic studies of memory to the characterization of more complex cognitive behavioral phenotypes.

## Data Availability Statement

The raw data supporting the conclusions of this article will be made available by the authors, without undue reservation.

## Ethics Statement

The animal study was reviewed and approved by University of Houston Institutional Animal Care and Use Committee.

## Author Contributions

JW, MS, CO’L, and JL designed the experiments and performed pilot studies. JW and TN performed the experiments. MS and JL analyzed the data and wrote the manuscript. All authors contributed to the article and approved the submitted version.

## Conflict of Interest

The authors declare that the research was conducted in the absence of any commercial or financial relationships that could be construed as a potential conflict of interest.

## Footnotes

1 www.craftparts.com
